# Elucidating the role played by bone marrow in visceral leishmaniasis

**DOI:** 10.3389/fcimb.2023.1261074

**Published:** 2023-10-04

**Authors:** Patricia Sampaio Tavares Veras, Maria Borges Rabêlo de Santana, Claudia Ida Brodskyn, Deborah Bittencourt Mothé Fraga, Manuela Silva Solcà, Juliana Perrone Bezerra De Menezes, Bruna Martins Macedo Leite, Helena Mariana Pitangueira Teixeira

**Affiliations:** ^1^ Laboratory of Parasite - Host Interaction and Epidemiology, Gonçalo Moniz Institute-Fiocruz Bahia, Salvador, Bahia, Brazil; ^2^ National Institute of Science and Technology of Tropical Diseases, National Council for Scientific Research and Development (CNPq), Salvador, Brazil; ^3^ Department of Preventive Veterinary Medicine and Animal Production, School of Veterinary Medicine and Animal Science, Federal University of Bahia, Salvador, Brazil

**Keywords:** visceral leishmaniasis, bone marrow, *Leishmania*, human visceral leishmaniasis, canine visceral leishmaniasis

## Abstract

Leishmaniasis is a widespread group of infectious diseases that significantly impact global health. Despite high prevalence, leishmaniasis often receives inadequate attention in the prioritization of measures targeting tropical diseases. The causative agents of leishmaniasis are protozoan parasites of the *Leishmania* genus, which give rise to a diverse range of clinical manifestations, including cutaneous and visceral forms. Visceral leishmaniasis (VL), the most severe form, can be life-threatening if left untreated. Parasites can spread systemically within the body, infecting a range of organs, such as the liver, spleen, bone marrow and lymph nodes. Natural reservoirs for these protozoa include rodents, dogs, foxes, jackals, and wolves, with dogs serving as the primary urban reservoir for *Leishmania infantum*. Dogs exhibit clinical and pathological similarities to human VL and are valuable models for studying disease progression. Both human and canine VL provoke clinical symptoms, such as organ enlargement, fever, weight loss and abnormal gamma globulin levels. Hematologic abnormalities have also been observed, including anemia, leukopenia with lymphocytosis, neutropenia, and thrombocytopenia. Studies in dogs have linked these hematologic changes in peripheral blood to alterations in the bone marrow. Mouse models of VL have also contributed significantly to our understanding of the mechanisms underlying these hematologic and bone marrow abnormalities. This review consolidates information on hematological and immunological changes in the bone marrow of humans, dogs, and mice infected with *Leishmania* species causing VL. It includes findings on the role of bone marrow as a source of parasite persistence in internal organs and VL development. Highlighting gaps in current knowledge, the review emphasizes the need for future research to enhance our understanding of VL and identify potential targets for novel diagnostic and therapeutic approaches.

## 1 Introduction

**Figure 1:**
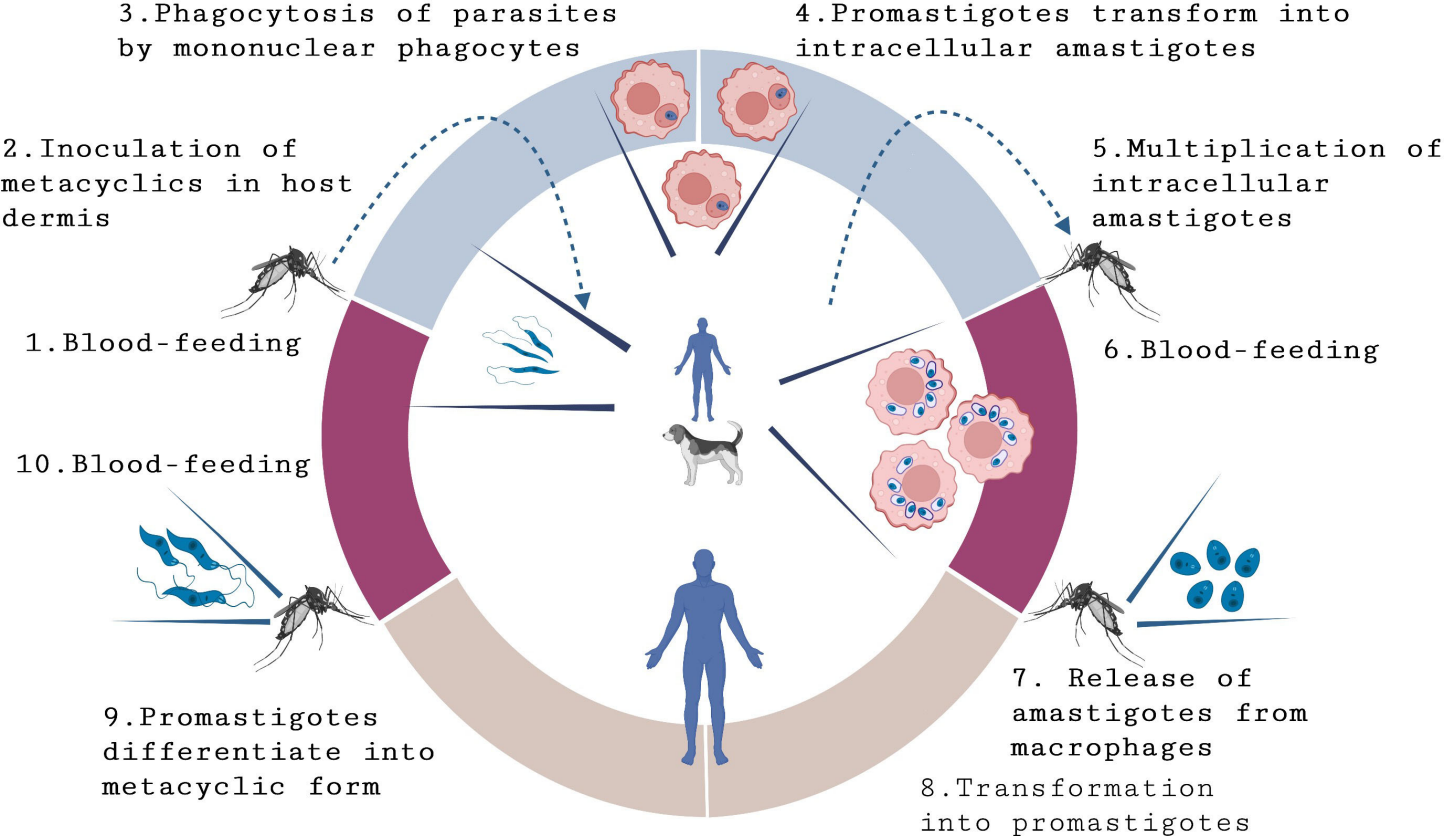
Life-cycle of *L. infantum* and *L. donovani*. Leishmaniasis transmission occurs through the bite of female phlebotomine sand flies that are carrying parasites. In the Old World, visceral leishmaniasis (VL) is anthroponotic, with *L. donovani* circulating among humans. However, in Europe, West Africa, and the Americas, VL is a zoonosis, primarily caused by *L. infantum*, which affects humans and dogs, which are considered as the main parasite reservoir. The transmission process begins when sand flies feed on blood (1) and inject the infective metacyclic form of *Leishmania* promastigotes (2) into the host’s dermis. Once inside the body, promastigotes are engulfed by macrophages (3) and other types of mononuclear phagocytic cells. Within these cells, promastigotes transform into amastigotes (4), the tissue stage of the parasite. Amastigotes multiply through simple division (5) and proceed to infect other mononuclear phagocytic cells, leading to the development and manifestation of the infection.

Leishmaniasis is a group of infectious diseases found on five continents, endemic in 102 countries, and estimated to be responsible for a loss of 2.53 million disability-adjusted life years (DALY) in 1990, 4.20 million DALY in 2009 to a reduced index of 0.98 million DALY in 2016 (Alvar et al., 2012; Ghatei and Haghdoost, 2013; Hay et al., 2017; Organization WH, 2019). Despite high prevalence, leishmaniasis is often neglected when prioritizing measures aimed at addressing tropical diseases (Organization WH, 2021). The causative agents of leishmaniasis are protozoan parasites of the genus *Leishmania* (Rangel et al., 2018), which produce a wide spectrum of clinical manifestations, ranging from the most common clinical form, cutaneous leishmaniasis (CL), to disfiguring mucocutaneous infections (ML) and visceral disease (VL) that harms internal organs and can be fatal if left untreated (BRASIL, 2014; Organization WH, 2019; Organization PAH, 2022a; Organization WH, 2023). VL reservoirs vary depending on geographic region and the specific *Leishmania* species involved (Alvar et al., 2012; Organization WH, 2021). In the Old World, Southeast Asia, and East Africa, where VL is an anthroponotic vector-borne disease, the etiological agent is *L. donovani* (Alvar et al., 2012; Talmi-Frank et al., 2012; Rock et al., 2016; Organization WH, 2021), while in Europe, West Africa, and the Americas, VL is considered a zoonosis, with *L. infantum* being the main species responsible for causing this clinical form (Alvar et al., 2004; Reis et al., 2006). *L. donovani* and *L. infantum* are protozoan parasites inoculated into the host dermis through the bite of insect vectors from the genera *Phlebotomus* and *Lutzomyia* during their blood meal. Within the host dermis, parasites infect mainly host macrophages and can spread systemically following propagation in these cells within internal organs, such as the liver, spleen, bone marrow, and lymph nodes (Kumar and Nylén, 2012). The control of leishmaniasis primarily involves treatment, which encompasses the administration of drugs like Pentavalent antimonials, Amphotericin B, and Miltefosin, often requiring extended courses of use (Croft and Olliaro, 2011). Rodents such as mice and rats, and other animals including dogs, foxes, jackals and wolves, serve as natural reservoirs of these protozoa (Quinnell and Courtenay, 2009). Dogs are considered the main urban reservoir of *L. infantum*. Due to clinical and pathological similarities between canine and human VL, dogs constitute an important model for the study of disease progression (Marzochi et al., 1985; Koutinas et al., 1999; Gontijo and Melo, 2004; Sanchez et al., 2004; Reis et al., 2006; Loría-Cervera and Andrade-Narváez, 2014) (**Figure 1**).

The clinical changes observed in both human and canine VL (CVL) include hepatomegaly, splenomegaly, lymphadenopathy, fever, weight loss, and hypergammaglobulinemia (Badaro et al., 1986). Additionally, hematologic abnormalities such as anemia, leukopenia with lymphocytosis, neutropenia, and thrombocytopenia have been documented (Sundar et al., 2008; Nicolato et al., 2013). Studies in dogs have linked these hematologic changes in peripheral blood to bone marrow alterations (Cotterell et al., 2000a; Abreu et al., 2011; Nicolato et al., 2013; Abidin et al., 2017). To further the understanding of hematologic and bone marrow changes with respect to disease pathogenesis, mouse models of VL have been instrumental in elucidating the mechanisms underlying these alterations.

The aim of this review article is to summarize relevant information regarding hematological and immunological changes, in addition to the persistence of parasites documented in the bone marrow of humans, dogs and mice infected with *Leishmania* spp. known to cause VL. Despite limitations, the animal models discussed in this review have been extensively used to study VL in an effort to better understand disease manifestations in humans. They, therefore, constitute valuable tools for advancing knowledge in this field and aiding in the identification of targets to develop new diagnostic and therapeutic approaches.

## 2 Epidemiology of VL

According to the World Health Organization (Organization WH, 2023), leishmaniasis ranks among the top ten neglected tropical diseases worldwide, being present on five continents and endemic in 102 countries, placing over 350 million people at risk of infection (Organization WH, 2023). Although human VL is endemic in 80 countries, the majority of cases occur in Brazil, Eastern Africa and India (Organization PAH, 2022a; Organization WH, 2023). In the Americas, VL has been described in at least 13 countries (Argentina, Bolivia, Brazil, Colombia, Costa Rica, El Salvador, Guatemala, Honduras, Mexico, Nicaragua, Paraguay, Uruguay, and Venezuela), with Brazil accounting for 93.5% of the cases reported in 2021 (BRASIL, 2022; Organization PAH, 2022b). From 2001-2010, an increasing trend in VL cases was observed throughout the Americas; however, from 2011-2021, this trend reversed, with 1,799 cases reported in 2021, the lowest number recorded in recent decades (Organization PAH, 2022b). Unfortunately, only 25 to 45% of new cases of human VL are reported to the WHO. Between 2001 and 2021, 69,665 new VL cases were notified, corresponding to an annual average of 2,488 cases and a fatality rate of almost 8% (Organization PAH, 2022a). However, according to the WHO, 5,710 deaths were attributed to VL alone in 2019 (Organization WH, 2023).

In Brazil, one of the countries in which VL is most prevalent (BRASIL, 2022; Organization PAH, 2022a), 21/26 states have reported cases across all five of the country’s regions (North, Northeast, Midwest, Southeast, South) (Brasil M da S, 1990). Over the last decade, the highest number of confirmed cases of human VL was 4,103 in 2017, leading to an incidence coefficient of 2.0 per 100,000 inhabitants. In contrast, 2021 was the year with the lowest number (1, 683) of reported cases (likely due to sub-notification during the pandemic) (Brasil M da S, 1990). The Northeast of Brazil is the most affected region, where the country’s main endemic areas are located. In 2017, 1,824 cases of VL were reported in this region, corresponding to 44% of the nationwide prevalence (Brasil M da S, 1990).

More recently, through a combination of targeted interventions, VL has been successfully controlled in certain countries as a result of actions, including improved access to diagnosis and treatment following sustained political commitments (Chapman et al., 2018; India Ministry of Health, 2018; Organization WH, 2021). In highly prevalent countries, such as India, a 50% reduction in VL incidence was recently reported compared to 2005 levels (India Ministry of Health, 2018). Other countries that have made progress in controlling VL include Bangladesh, Nepal and Brazil (Chapman et al., 2018; Rangel et al., 2018). However, despite these successful efforts, VL remains a significant public health challenge due to high mortality in many parts of the world, particularly in East Africa, where endemicity continues to lead to outbreaks (Organization WH, 2021).

With respect to canine visceral leishmaniasis (CVL), estimating worldwide prevalence presents challenges, as data are collected from several independent studies, yet not all countries and endemic regions have been adequately characterized. However, it is notable that the presence of infected dogs has been linked to increases in human VL cases in many endemic areas (Simões-Mattos et al., 2005; Araújo et al., 2013; Barbosa et al., 2014). In addition, CVL has seemingly grown in parallel to human VL across several parts of the world, contributing to the recognition of dogs as the main reservoir of parasites in urban areas (Alvar et al., 2004; BRASIL, 2014).

In endemic areas of Brazil, the Ministry of Health has estimated that each case of human VL corresponds to at least 200 infected dogs, leading to an estimated 700,000 cases of CVL annually (da Saúde Mato Grosso do Sul BRASIL M, 2020). CVL distribution extends beyond the most prevalent Northeastern region to others, such as the Southeast, with expanding numbers of cases reported in Rio de Janeiro (Júnior et al., 2015), Minas Gerais (Nunes et al., 2016; Ursine et al., 2016) and São Paulo (Oliveira et al., 2016) in recent years. In the South, the first cases of CVL were reported in 2008 in Rio Grande do Sul, which preceded the appearance of the first cases recorded in humans in 2009 in the same region (Da Silva et al., 2011; Júnior et al., 2015).

## 3 Hematological alterations in peripheral blood during VL

Although less prevalent than cutaneous leishmaniasis, VL can be fatal if left untreated, and is potentially deadly in children aged five years or younger (Organization WH, 2021). The severity of the disease can vary widely depending on factors such as age, as well as immune and nutritional status. Common VL manifestations in humans include prolonged fever, weight loss, hematological alterations, such as anemia and thrombocytopenia, enlargement of spleen and liver, and hypergammaglobulinemia. In some cases, patients may present severe complications, such as bleeding disorders and renal failure (Murray et al., 2003) (**Table 1**).

**Table 1:** Hematological abnormality prevalence in VL patients.

Hematological Abnormality	Prevalence Range in VL Patients	References
Anemia	83.8% to 97.4%	(Sarkari et al., 2016; Mulaw et al., 2018; Shiferaw et al., 2021; Debash et al., 2023,)
Anemia in Children	More severe	(Al-Jurayyan et al., 1995)
Thrombocytopenia	40% to 91.2%	(Rahim et al., 1998; Queiroz et al., 2004; Chakrabarti et al., 2013; Chufal et al., 2016; Shiferaw et al., 2021; Debash et al., 2023)
Leucopenia	Varies in different studies	(Shiferaw et al., 2021; Debash et al., 2023)
Neutropenia	74.2% to 82.5%	(Shiferaw et al., 2021; Debash et al., 2023)
Pancytopenia	71.2% to 79.4%	(Negera et al., 2008; Grifferty et al., 2021; Debash et al., 2023)

Anemia is a common hematological alteration in the peripheral blood seen in patients with VL, frequently from a normochromic normocytic type. The prevalence of anemia reportedly ranges from 83.8% to 97.4% in patients with VL (Sarkari et al., 2016; Mulaw et al., 2018; Shiferaw et al., 2021; Debash et al., 2023). Children affected by this disease tend to experience more severe anemia (Al-Jurayyan et al., 1995). Anemia severity has been associated with various factors, including red blood cell hemolysis, malnutrition, the presence of additional comorbidities, such as chronic illness, and opportunistic infection (Tesfaye et al., 2017).

In VL, the anemia can be caused by the destruction of red blood cells by the parasite or due to bone marrow suppression resulting from infection. Anemia may also be caused by other mechanisms, including the sequestration and destruction of red blood cells in an enlarged spleen, immune changes, alterations in the membrane permeability of red blood cells, and increased plasma volume in VL patients (Varma and Naseem, 2010; Neki and Singh, 2017). Hemophagocytosis, a frequent finding in studies on VL patients (Gagnaire et al., 2000; Özyürek et al., 2005; Kilani et al., 2006; Bhatia et al., 2011; Chandra et al., 2013), may also contribute to anemia, in addition to dyserythropoiesis and ineffective erythropoiesis, which may be caused by the effects of toxins produced by the parasite or other mechanisms (Wickramasinghe et al., 1987). Although dyserythropoiesis in VL is a well-known condition, few cases detailing this finding have been reported in the literature (Olivieri et al., 1987; Shahriar et al., 1999; Bain, 2010). Since myelodysplastic syndrome is relatively common in adults, this may lead to some confusion in the diagnosis of leishmaniasis, particularly in cases lacking the classical feature of parasite detection in bone marrow (Bain, 2010). It follows that differentiating features should be considered to aid in VL diagnosis and treatment.

Another frequently observed alteration is thrombocytopenia, characterized by a significant prevalence of reduced platelet counts ranging from 40% to 91.2% in patients with VL (Rahim et al., 1998; Queiroz et al., 2004; Chakrabarti et al., 2013; Chufal et al., 2016; Shiferaw et al., 2021; Debash et al., 2023). In an apparent contradiction, another study did not find correlations between thrombocytopenia and parasite index or leukopenia (Hamid and Gobah, 2009). The variability in the prevalence of this alteration may be attributed to differences in sample size, classification criteria, and study design. This sign has been mainly attributed to splenic sequestration and immune-mediated processes since about one-third of human platelets are stored in the spleen and any abnormalities in this organ can lead to reductions in platelet counts (Neki and Singh, 2017). This sign can also result from bone marrow suppression and hepatomegaly associated with disease progression (Shiferaw et al., 2021).

To better understand the mechanism underlying thrombocytopenia in VL, Rani et al. (2021) used a murine model of C57BL/6 mice infected with *L. donovani* and investigated megakaryocytes alterations associated with VL (El-Hassan et al., 1990). The authors reported that *L. donovani* infection provoked a progressive reduction in platelet counts, resulting in severe thrombocytopenia by day 28. The observed decrease in platelets resulted from multiple factors, including reduced plasma thrombopoietin (TPO) levels, alterations in the liver microenvironment due to granulomatous inflammation, and significant increases in platelet clearance. Additionally, infected mice presented higher levels of platelet opsonization and desialylation, which were associated with platelet clearance in the spleen and liver, respectively. Interestingly, these changes were quickly reversed by reducing parasite load via drug treatment or by the administration of TPO agonists, which indicates that the mechanisms behind thrombocytopenia in *L. donovani*-infected mice are multifactorial and reversible. The authors also proposed that platelet counts could be a valuable tool for measuring disease progression or response to treatment in both real-life infections, as well as experimental models (El-Hassan et al., 1990).

Leucopenia has also been reported as a hematological abnormality in some studies. The prevalence of neutropenia has been reported to vary from 74.2% to 82.5% in VL patients from Ethiopia (Shiferaw et al., 2021; Debash et al., 2023). As *Leishmania* parasites can cause damage to immature white blood cells, especially neutrophils, this may lead to the high observed prevalence of neutropenia (Neki and Singh, 2017). Increased neutropenia in children with VL has been strongly associated with massive splenomegaly (El-Hassan et al., 1990).

The general reduction in the numbers of red blood cells, white blood cells and platelets, known as pancytopenia, is another common finding in VL, which can lead to an increased risk of bleeding and infections. The prevalence of pancytopenia reportedly ranges from 71.2% to 79.4% (Negera et al., 2008; Grifferty et al., 2021; Debash et al., 2023).

In addition to being considered the main reservoir for *L. infantum*, dogs also constitute a valuable model for studying disease progression, since the clinical manifestations observed in CVL resemble those evidenced in human VL patients (Marzochi et al., 1985; Koutinas et al., 1999; Gontijo and Melo, 2004; Sanchez et al., 2004; Reis et al., 2006; Loría-Cervera and Andrade-Narváez, 2014). Infected animals exhibit a range of presentations, from mild to severe or even disseminated forms of the disease. While the progression and containment of VL in dogs is not yet fully understood, it has been postulated that several factors are involved, including parasite virulence, host genetic factors, and the type of immune response triggered after infection (Reiner and Locksley, 1995).

**Figure 2:**
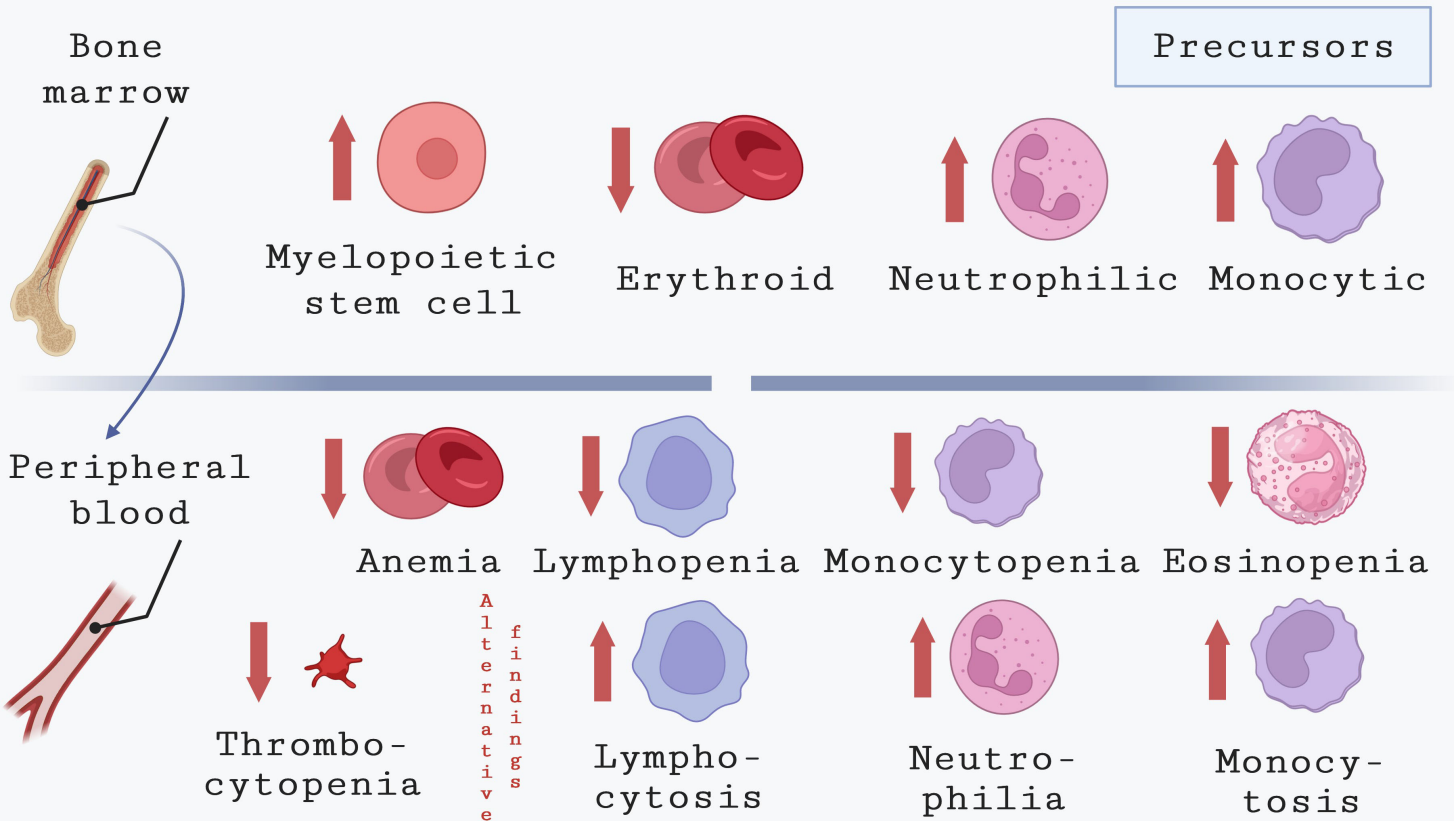
Hematological alterations in bone marrow and peripheral blood during CVL. This image depicts the common hematological changes observed in the peripheral blood of dogs infected with *L. infantum*. Although the underlying causes appear to differ from those in humans, dogs with CVL commonly exhibit anemia along with thrombocytopenia, eosinopenia, and lymphopenia. By contrast, leukocytosis associated with neutrophilia and monocytosis in CVL was also described in CVL.

The clinical characteristics of CVL can vary widely, including weight loss, lethargy, anorexia, and fever, in addition to skin lesions, such as alopecia, onychogryphosis and renal insufficiency. Like humans, hematological alterations are also commonly observed in dogs with visceral disease (Nicolato et al., 2013; De Tommasi et al., 2014; Maia and Campino, 2018; Meléndez-Lazo et al., 2018) (**Figure 2**) Anemia is a common alteration in dogs with CVL. This alteration probably originates from several factors frequently associated with chronic inflammatory diseases (Reis et al., 2006; Paltrinieri et al., 2016), such as defective erythropoiesis due to decreased erythropoietin production in animals presenting renal failure. Furthermore, anemia in CVL can also occur as a less common cause, specifically as a result of increased eryptosis and macrocytic hypochromic regenerative anemia (Maia and Campino, 2018).

Another common hematological alteration related to the chronicity of CVL is the neutrophilia, which has been associated with parasite persistence in different organs of symptomatic dogs (Almeida et al., 2021). In some endemic areas, many affected animals are strays that are exposed to multiple tick-borne pathogens, including *Ehrlichia canis*, *Anaplasma platys* and *Babesia vogeli*, all of which can cause neutrophilia (Alvar et al., 2004; de Sousa et al., 2013). Other hematological alterations, such as monocytopenia, eosinopenia and lymphopenia, are also considered common findings in the blood of dogs with CVL (Reis et al., 2006; Nicolato et al., 2013; Torrecilha et al., 2016; Meléndez-Lazo et al., 2018). Contradictory, other reports documented leukocytosis associated with neutrophilia and monocytosis in CVL (Ciaramella et al., 2005; Terrazzano et al., 2006; Ikeda-Garcia et al., 2008; Almeida et al., 2021).

Bleeding and thrombocytopenia have been described as common signs in dogs naturally infected with *Leishmania* of the donovani complex (Sanchez et al., 2004; Baneth et al., 2008; Koutinas and Koutinas, 2014; Paltrinieri et al., 2016). However, Meléndez-Lazo et al. (2018) comprehensively investigated clinical and pathological alterations in dogs naturally infected with *L. infantum*, reporting both thrombocytosis and thrombocytopenia as common platelet alterations, yet with the notable absence of bleeding (Meléndez-Lazo et al., 2018). These discrepancies could potentially stem from variations in canine populations, as elucidated earlier. In certain regions endemic to VL, a considerable number of infected animals are strays, and they might come across significant exposure to tick-borne pathogens responsible for diseases that manifest with bleeding symptoms. The simultaneous occurrence of those diseases and leishmaniasis could enhance the hematological changes seen in dogs with VL. While the precise mechanisms responsible for bleeding disorders in infected dogs require additional investigation, Terrazzano and collaborators (2005) demonstrated an association between anti-platelet IgM and IgG antibodies in the serum of dogs and VL pathogenesis (Terrazzano et al., 2006).

Additional abnormalities related to blood clotting have been reported in dogs with CVL, including elevated serum viscosity, thrombocyte dysfunction, impaired clotting factor activity and fibrinolysis, excessive fibrinogen levels, as well as elevated prothrombin and activated partial thromboplastin times (Ciaramella et al., 2005; Petanides et al., 2008; Paltrinieri et al., 2010).

## 4 Bone marrow alterations during VL

Bone marrow is a complex microenvironment containing various cell types, including stem and progenitor cells, hematopoietic cells, non-hematopoietic endothelial cells, stromal cells, and immune cells that together are known as stroma.

Bone marrow samples are commonly used for detecting parasites in the diagnosis of VL, yet parasite detection is not always possible, particularly in regions where the disease is not endemic. To overcome this limitation, Rodriguez and colleagues (2019) established a method based on bone marrow alterations observed through microscopic examination, used as a tool for diagnosing VL (Chufal et al., 2016; Lockard et al., 2019). Increases in erythroid series, plasma cells, macrophages and megakaryocytes, decreases in myeloid series and the myeloid-erythroid ratio, as well as hemophagocytosis of platelets and leukocytes were found to apparently distinguish patients with VL from those with suspected disease (de Mello Deane, 1958; Marzochi et al., 1985; de Sousa et al., 2013; Varjão et al., 2021). Hypercellularity was also found to be a commonly reported bone marrow alteration found in 90% of children that tested positive for the presence of parasites from a population enrolled to perform morphological analysis under optical microscopy in sub-Himalayan regions of India (Wickramasinghe et al., 1987). As in other studies, the authors also found frequent presence of erythrophagocytosis, leukophagocytosis, and increased numbers of histiocytes. Less commonly finding observed in their bone marrows was granulomatous formation, similar to a previous case report by Finocchi and colleagues (2008), which described in the bone marrow of a patient no alterations in red blood cell lineage, despite intense hypercellularity due to granuloblast hyperplasia (Finocchp et al., 2008) (Kumar et al., 2007). Interestingly, hypercellularity in combination with the presence of benign lymphoid nodules in bone marrow was associated with a better prognosis, in children from Iran, as these patients evolved to cure following Glucantime treatment. Conversely, patients with bone marrow fibrosis and necrosis remained unresponsive to both forms of therapy (Kumar et al., 2007).

The mechanisms that underlie this apparent discrepancy between the reduction of blood cells on the periphery associated with hypercellularity in the bone marrow need further investigation. Disease chronicity and splenomegaly may induce pancytopenia, leading to high rates of peripheral blood cell death (Varma and Naseem, 2010). Some reports have described patients with VL also exhibiting peripheral blood lymphocytopenia (Al-Ghazaly et al., 2017) with bone marrow lymphocytosis. In an attempt to explain the coexistence of peripheral blood lymphocytopenia and bone marrow lymphocytosis, it has been postulated that lymphocytes first migrate to the infected lymphoid tissues to mount an inflammatory response, while bone marrow lymphocytosis presents as a compensatory response to supply lymphocytes to organs affected by the parasite (Bourdoiseau et al., 1997; Reis et al., 2006).

**Figure 3:**
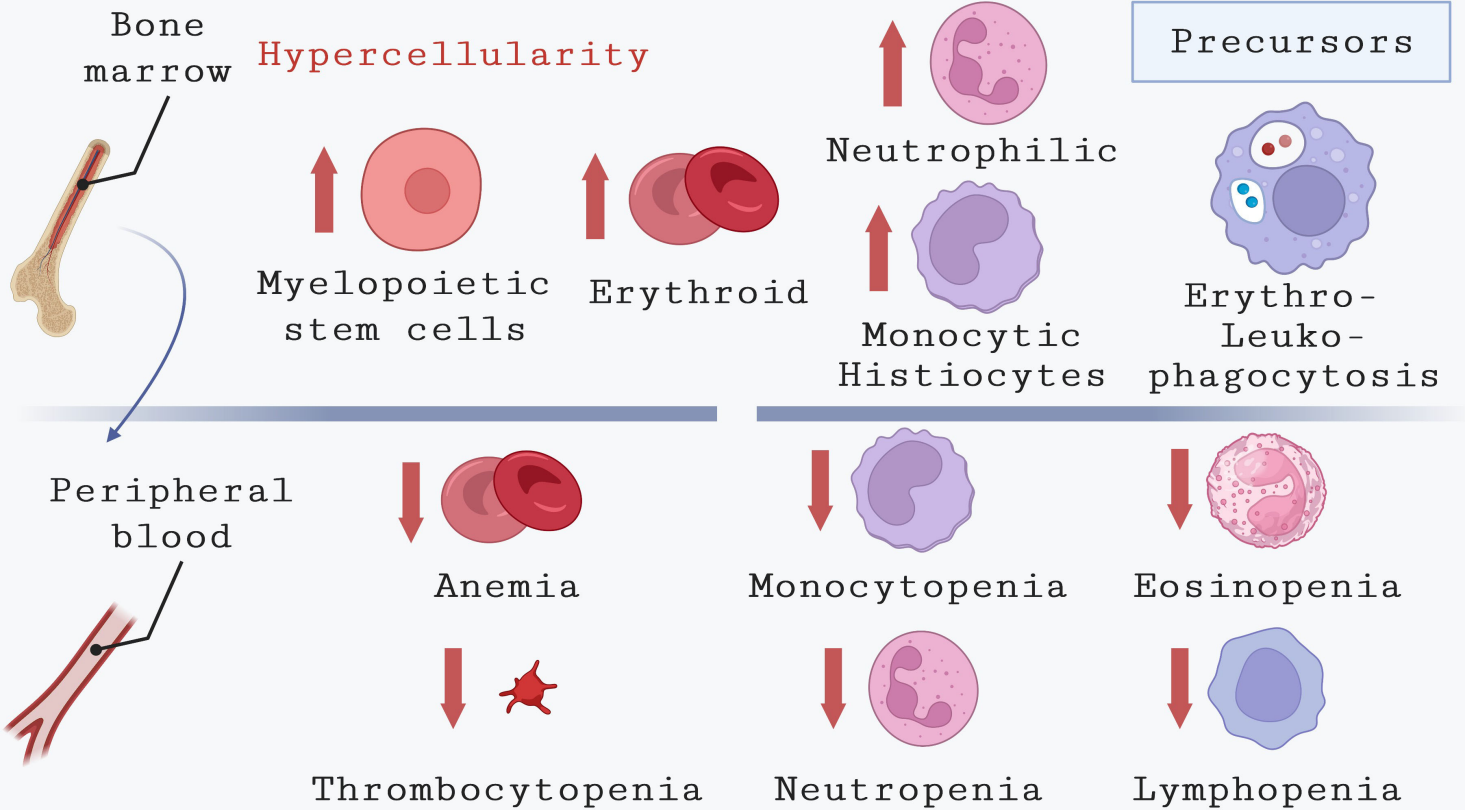
Hematological alterations in bone marrow and peripheral blood during VL. This illustration highlights the main hematological abnormalities observed in the peripheral blood of humans affected by VL. These include anemia, which can result from various causes, thrombocytopenia, and pancytopenia due to reduced counts of monocytes, neutrophils, eosinophils, and lymphocytes. The reduction in cell counts coexists in bone marrow with an increase in cells from different lineages, such as myelopoietic stem cells, erythroid precursors, neutrophilic precursors, and monocytic precursors. Additionally, common alterations observed in the bone marrow of VL patients include erythrophagocytosis and leukophagocytosis.

Regarding the role played by bone marrow on VL outcome, hematological alterations found in bone marrow (**Figure 3**), including hemophagocytosis and granulomatous lesions associated with hypersplenism, chronic inflammation, and dietary factors may influence the pathogenesis of this disease (Al-Jurayyan et al., 1995; de Sousa et al., 2013; Safi et al., 2016; Segarra et al., 2018). In addition, it has been suggested that the central role of this tissue in the pathogenesis of VL may be due to its high susceptibility to *L. infantum*, with myeloid cells being one of the primary host cells where parasites are found (Baldridge et al., 2010; Lafuse et al., 2013; Manz and Boettcher, 2014; Matatall et al., 2014). Infection and replication of parasites within the bone marrow led to alterations in the number of peripheral blood cells, resulting in anemia, thrombocytopenia, and leukopenia.

In a cross-sectional study conducted by Almeida et al. in 2021, a comparative analysis was performed to examine the changes in bone marrow and peripheral blood in dogs naturally infected with *L. infantum* (Almeida et al., 2021). This study revealed that dogs exhibiting clinical signs of VL, different from humans, showed erythroid hypoplasia as the primary bone marrow alteration (Alvar et al., 2004; de Sousa et al., 2013). This finding correlated with lower levels of erythrocytes, hemoglobin, and hematocrit in the peripheral blood. Also in dogs, monocytopenia, and lymphopenia are described as common findings in the peripheral blood of dogs with CVL (Koutinas et al., 1999; Sanchez et al., 2004; Torrecilha et al., 2016; Meléndez-Lazo et al., 2018). By contrast, it was, recently, described that infected dogs with active disease showed leukocytosis due to increased numbers of neutrophils and monocytes compared to uninfected dogs (Almeida et al., 2021)(**Figure 2**).

While hypersplenism has been considered the principal cause of pancytopenia in humans with VL, resulting from the trapping and removal of blood cells from the periphery, the study suggested that hypoplasia of the erythroblastic series in the bone marrow can be the primary cause of anemia in dogs with severe VL. This implies an abnormality in cell production rather than solely spleen-related factors. Almeida et al. proposed that the hematological alterations observed in dogs may stem from imbalanced communication between hematopoietic cells derived from pluripotent cells in the bone marrow and other components in the bone marrow stroma. This imbalance could hinder the differentiation process, preventing precursor cells from reaching the peripheral blood (Almeida et al., 2021).

Nonetheless, the contribution of the spleen in hematological alterations cannot be overlooked. Dogs with splenic disorganization due to *L. infantum* infection exhibited more pronounced hematological abnormalities, including anemia, peripheral blood leukocytosis with neutrophilia, and erythroid hypoplasia in the bone marrow, compared to dogs with organized spleens (Almeida et al., 2021).

## 5 Influence of *L. infantum* on the immune response originating in bone marrow

As described above, bone marrow stroma is formed by various cell types, including stem and progenitor cells, hematopoietic cells, non-hematopoietic endothelial cells, stromal cells, and immune cells that together are crucial for the mounting of an effective immune response against parasites, as shown by studies conducted by Ribeiro-Gomes et al. (2012) and Cotterell et al. (2000) (Cotterell et al., 2000b; Ribeiro-Gomes and Sacks, 2012).

Protective immunity against *Leishmania* relies on the interaction between innate and adaptive host immune responses. The recognition of *Leishmania* parasites by myeloid immune cells is essential for initiating an effective immune response. However, these initial interactions can lead to varying outcomes, either the elimination or the persistence of parasites within the host myeloid cells. Consequently, infection in the bone marrow can impact immune responses and induce the release of cytokines and chemokines that have the potential to affect the progression of diseases (Martínez-López et al., 2018).

Although neutrophils are the first cells that migrate to the site of parasite inoculation, the role played by these cells in VL pathogenesis remains largely unknown, as they can either promote or protect against infection. Yizengaw and colleagues (2016) discovered that individuals with VL exhibit a significant degree of neutrophil activation and degranulation, which is consistent with the inflammatory characteristics of this disease (Yizengaw et al., 2016). Beyond activation and degranulation, which correlate with the inflammatory characteristics of VL, neutrophils can also produce extracellular traps (NETs) in response to different *Leishmania* species, including *L. donovani* and *L. infantum*. The generation of NETs could play a significant role in controlling and, consequently, impacting disease progression. The parasite triggers the release of NETs from neutrophils in both healthy and those naturally *Leishmania infantum*-infected dogs. Moreover, the roles of phagocytosis and NETs in eliminating parasites demonstrate distinct dynamics in neutrophils from healthy and infected animals. Furthermore, the parasite differentially influences the modulation of IFN-γ, IL-8, IL-4, and TNF-α production by neutrophils in both groups (Wardini et al., 2019).

Monocytes/macrophages are considered the most crucial cells in disease development, as these are the cells in which parasites primarily reproduce. Conversely, once properly activated, these cells can be also responsible for parasite elimination contingent upon the development of an adaptive Th1 immune response via specific dendritic cell subtypes (Martínez-López et al., 2018). One of the first reports on adaptive immunity originating in bone marrow cells focused on the characterization of lymphocyte subsets in the bone marrow of patients with either acute or chronic VL (Rohtagil et al., 1996). The authors concomitantly identified higher CD4^+^ T cell counts and lower CD8^+^ T cell counts in bone marrow; following antileishmanial therapy, a significant increase in CD8^+^ T cell counts was observed. The observed rise in this lymphocyte population in both the bone marrow and spleen was associated with a proportional reduction in CD4^+^ T cell populations, which occurred due to migration from the reticuloendothelial organs to peripheral blood (Rohtagil et al., 1996).

In a more recent study, Cotterell and colleagues employed a BALB/c mouse model infected with *L. donovani* to thoroughly investigate the potential mechanisms that underlie the immune response originating from the bone marrow and its impact on hematological alterations in VL (Cotterell et al., 2000b). The specific type of cytokines secreted is known to vary between hosts, as well as among different animal models.The role played by proinflammatory cytokines in VL has been consistently demonstrated, as interferon (IFN)-γ, interleukin (IL)-12, IL-2 and tumor necrosis factor (TNF) are known to play an important role in macrophage activation and parasite killing within these cells (Martínez-López et al., 2018).”

These authors described that monocytes derived from bone marrow play a crucial role in effectively eliminating parasites from the organ, and they are likely activated to a leishmanicidal state due to the predominance of Th1 cytokines in the granuloma environment. In contrast to the liver, where parasites are initially contained, in the spleen and bone marrow parasites persist and their expansion in these organs is associated with increased parasite numbers. During this critical phase of infection, there is a significant increase in the numbers and proliferative activity of hematopoietic progenitor cells in both the spleen and bone marrow (Sundar et al., 2008).

Produced in response to immune cell activation arising from *Leishmania* infection, cytokines are believed to play a critical role in determining VL outcome. Recently, Samant et al. (2021) have elegantly delineated the differences found in cytokine production and function between human subjects and animal models (Samant et al., 2021).

The specific role played by bone marrow monocytes in both the progression of human VL and response to therapy has been recently addressed using a C57BL/6 mouse model infected with *L. donovani*. The authors focused on bone marrow Ly6C^hi^ inflammatory monocytes which, once activated, can migrate to the site of infection in response to inflammation (Al-Jurayyan et al., 1995; Agrawal et al., 2013; Chakrabarti et al., 2013; Chandra et al., 2013; Chufal et al., 2016; Lockard et al., 2019). In Wild Type (WT) C57BL/6 mice, *L. donovani* infection drove CD4^+^ T cells to produce IFN-γ. Ly6Chi monocyte activation in *L. donovani*-infected C57BL/6 mice was limited by IL-10, which is also produced by CD4+ T cells. The blockade of IL-10 resulted in faster infection resolution, as the intensity of IFN-γ production by CD4+ T cells was observed to correlate with healing in IL-10-/- mice. In addition, the authors also found *L. donovani* parasite load to be directly linked to immune activation. They then concluded that the interplay between IFN-γ, IL-10 and parasite load plays a critical role in the activation of bone marrow monocytes, which is highly linked to the outcome of *L. donovani* infection in mice (Chakrabarti et al., 2013; Fampa et al., 2021).

Based on insights gleaned from the C57BL/6 mouse model, it has been documented that active VL in humans is characterized by a strong inflammatory response. A recent study identified elevated human serum levels of several inflammatory biomarkers, including Prostaglandin F2α (PGF2α), Leukotriene B4 (LTB4), Resolving D1 (RvD1), IL-1β, IL-6, IL-8, IL-10, IL-12p70, and TNF-α (Lima et al., 2017). The authors also noted decreased levels of TGF-β1 in VL patients compared to uninfected controls residing in the same endemic area. *Leishmania* treatment was observed to reverse these alterations, indicating that the targeting of these inflammatory pathways could represent a potential strategy for host-directed therapy against VL (Lima et al., 2017).

Immunosuppression has also been observed in symptomatic patients during later stages of infection, which can be correlated with the presence of IL-10 produced in response to IFN-γ (Shahriar et al., 1999; Varma and Naseem, 2010; Fampa et al., 2021; Patel et al., 2021) Kumar and colleagues (2018) investigated the relationship between parasite load as a marker of disease severity and T cell function in bone marrow (Kumar et al., 2007; Kumar et al., 2018). In a sample of 26 VL patients from India, individuals presenting higher parasite load also had increased frequencies of IL-10-producing T regulatory (Foxp3+) cells. In contrast, the proportions of effector cells producing IL-17 and IFN-γ were lower in these same patients. Blocking IL-10 and TGF-β *in vitro* led to enhanced effector cell function, as evidenced by greater IFN-γ and IL-17 production by the patient’s lymphocytes. This finding indicates that IL-17-producing activated CD4+ T cells, differentiated from a functional subpopulation of T cells (Normanton and Marti, 2013), play a potential protective role in VL (Banerjee et al., 2016). In addition, a high IL-6/TGF-β ratio favors the pro-inflammatory cytokine IL-6 over TGF-β, thereby contributing to the increased inflammatory activity observed in VL patients that could be also protective in this case (Samant et al., 2021).

Recently, Almeida and colleagues (2021) conducted a study on CVL and observed that dogs naturally infected with *L. infantum* showed elevated gene expression levels of both IFN-γ and TNF in the bone marrow (Segarra et al., 2018; Almeida et al., 2021). Interestingly, the presence of *L. infantum* in the bone marrow did not cause any changes in the gene expression levels of other cytokines such as TGF-β, IL-1β, or IL-10. Furthermore, IL-4 was not detected in the collected bone marrow samples from the studied dogs. These findings appear to be contradictory to the immunological changes observed in human and experimental VL, where cytokines, such as IL-10, TGF-β, and IL-6 secreted in the periphery have been associated with disease progression (Samant et al., 2021). Therefore, it is mandatory to develop studies to further investigate the actual cytokine production that takes place in the bone marrow during the establishment and progression of the disease considering various models of VL. For instance, descriptions regarding alterations in cytokine production in the peripheral immune system have greatly enhanced our understanding of the immunological response that contributes to VL pathogenesis.

In dogs, the direct impact of the immune response originating in the bone marrow on the outcome of *Leishmania* infection also has not been clearly elucidated. However, a recent cohort study of dogs conducted in an endemic region for VL within the municipality of Camaçari (Bahia-Brazil) investigated immunological parameters and clinical signs of disease progression in dogs with CVL. After classifying animals as either resistant or susceptible to infection, the authors found that susceptible dogs presented higher splenic parasite load and greater parasite persistence compared to resistant dogs (Martínez-López et al., 2018; Segarra et al., 2018). However, no statistically significant differences in serum levels of IFN-γ, IL-8, CCL2 or IL-10 were found during follow-up among resistant and susceptible groups. Also, in an attempt to gain insight into the dynamics of immune response, the authors sought to identify interactions between biological mediators within each group of dogs at different time points throughout the two-year study period. Interactome networks identified differences in immune response dynamics at two-time points (before and after the infection diagnosis) in both CVL-susceptible as well as CVL-resistant dogs. A significant decrease in the number of correlations involving IFN-γ detected after infection was diagnosed in susceptible dogs, whereas similar participation of IL-10 was observed both before and after infection. By contrast, in resistant animals, decreased involvement of both IFN-γ and IL-10 was observed in biological networks following diagnosis (Varjão et al., 2021).

It has been demonstrated that other organs, besides peripheral blood, can impact bone marrow response secondary to *Leishmania* infection. The vital role of the spleen in protecting against bacterial infections has been clearly described. As described above, only recently it was shown that dogs naturally infected with *L. infantum* with susceptibility to VL exhibit spleen disorganization, which was found to negatively impact the bone marrow compartment, as evidenced by the sharp levels of IFN-γ and TNF gene expression in the bone marrow of infected animals (Santos et al., 2008; Araújo-Santos et al., 2017; Almeida et al., 2021). Evidence of an indirect effect of the spleen on bone marrow response further emphasizes the crucial role of this organ in providing protection against VL.

## 6 Bone marrow and parasite persistence within the host

Although alterations in hematopoiesis are commonly evidenced in experimental models of infectious disease, few studies have attempted to elucidate the mechanisms underlying hematopoietic alterations arising from *L. donovani*, in which parasites disseminate to different lymphoid organs (Cotterell et al., 2000b). Cotterell et al. (2000) demonstrated that intracellular parasitism in a stromal cell population may hamper the regulation of hematopoiesis, highlighting the enhanced capacity of stromal macrophages to directly support myelopoiesis *in vitro* (Cotterell et al., 2000b). This effect was mediated through the induction of granulocyte-macrophage colony-stimulating factor (GMCSF) and TNF production. As explained above, according to these researchers, it was noted that parasites persist in the spleen and bone marrow, and their growth in these tissues is connected with an increase in parasite number. In this pivotal infection stage, there is a notable rise in the quantities and replication rate of hematopoietic precursor cells within both the spleen and bone marrow, providing a possible explanation for the extended course of the disease (Sundar et al., 2008). The correlation between this heightened hematopoietic activity and the expansion of parasites in the spleen and bone marrow suggest a potential relationship between these two events. A more recent study by Abidin (2018) revealed an expansion in Sca1+ mesenchymal stem cells (MSC) in the bone marrow of BALB/c mice infected with *L. donovani*, with most of these cells being Ly6C^hi^ monocytes displaying a regulatory, suppressor-like phenotype (Abidin et al., 2017). Furthermore, the properties exhibited by these cells reinforce the notion that MSCs provide a favorable environment for *L. donovani* persistence in the host delaying VL cure. This class of MSCs exhibits self-renewal capability, low levels of oxidative stress in response to reactive oxygen species, as well as low expression of MHC Class II and MHC Class I molecules with effector functions (Abidin et al., 2017). The role played by these cells in maintaining VL persistence offers a compelling avenue to advance our comprehension of the mechanisms underlying treatment failure.

It was recently shown, using an alternative experimental model of VL, that *L. infantum* promastigotes also effectively infect CD271^+^/Sca1^+^ bone marrow MSCs derived from C57BL/6 mice both *in vitro* and *in vivo*. The high expression of drug efflux pumps in these MSCs may be related to *Leishmania*’s ability to evade drug effects (Lopes et al., 2016). Moreover, another study demonstrated that several *Leishmania* species, including those causing cutaneous leishmaniasis and VL, can persist in an inactive form in cultures of MSCs derived from adipose tissue (Allahverdiyev et al., 2011). As stem cells tend to remain quiescent in the absence of an external stimulus, this suggests that they may also serve as an ideal host cell reservoir for *Leishmania* (Allahverdiyev et al., 2011). While the mechanism by which *Leishmania* invades MSCs has yet to be fully elucidated, it has been reported that the MSCs found in adipose tissue possess phagocytic properties (Valigurová and Kolářová, 2023).

More complex interactions between hosts and parasites result from the introduction of an inflammatory challenge via LPS in malnourished mice. It was recently shown that such an inflammatory stimulus promoted the expansion of MSCs and bone marrow adiposity in malnourished animals infected with *L. donovani*. Although the authors did not evaluate the impact of this stimulus on infection persistence, they did show that bone marrow MSCs differentiated towards an adipocyte lineage instead of bone-forming osteoblasts (Osorio et al., 2022). As the study demonstrated inflammasome activation together with a high proportion of inflammatory monocytes expressing IL-1β, it is therefore possible that parasite load may be controlled in inflamed tissue following priming with LPS (Osorio et al., 2022).

Regarding parasite persistence in organs such as the bone marrow, the discontinuation of primary VL treatment regimens was hypothesized as a model capable of elucidating associations between therapeutic failure and disease relapse (Valigurová and Kolářová, 2023). Recently, Dirkx et al. (2022) employed double-bioluminescent/fluorescent *L. infantum* or *L. donovani* as reporter lines, in *L. infantum*-infected BALB/c mice, and identified long-term hematopoietic stem cells (LT-HSCs) as a niche with high parasite burden in bone marrow, a feature also seen in human hematopoietic stem and progenitor cells (hHSPC) (Dirkx et al., 2022). LT-HSCs were shown to be more tolerant to antileishmanial drugs and were thus identified as a source of relapse. In these cells, the authors detected a unique transcriptional signature characterized by the upregulation of both TNF/NF-κB and RGS1/TGF-β/SMAD/SKIL signaling, in conjunction with the downregulation of oxidative burst (Dirkx et al., 2022).

While it is generally accepted that bone marrow alterations occur during VL, a recent study examined infection-adapted myelopoiesis in a chronic experimental murine model of cutaneous leishmaniasis (CL) caused by *L. major* (Ferreira et al., 2022). Previous studies had shown that the activation of HSPCs promotes parasite persistence in experimental VL by increasing the production of permissive monocytes (Cotterell et al., 2000a). Interestingly, the more recent study evidenced an expansion of myeloid-biased HSPC in the bone marrow and spleens of mice infected with a persistent strain of *L. major*, despite the absence of viable parasite detection in the bone marrow. Additionally, these authors observed an increase in monocytes and monocyte-derived myeloid cells in the spleen. Furthermore, mice infected with this persistent strain exhibited a diminished type I/type II interferon response compared to a self-healing strain, although both strains induced a rapid upregulation of myelopoietic cytokines, such as IL-1β and GM-CSF. These results suggest that the presence of *Leishmania* parasites in the bone marrow may not be essential for infection-adapted myelopoiesis in the CL model, which further highlights the importance of studying bone marrow alterations during the course of both VL and CL infections

## 7 Conclusion

This review emphasizes the crucial role of the bone marrow in the hematological and immunological responses observed in peripheral tissues during *Leishmania* infection, particularly in the context of visceral leishmaniasis (VL). These responses are believed to directly influence the development of VL. The review suggests that further studies using animal models should be conducted to gather complementary data, address limitations, and improve our understanding of the mechanisms underlying the apparently contradictory alterations observed in the bone marrow of humans, dogs, and mice following infection. By achieving a deeper comprehension of the interactions between *Leishmania* parasites and the bone marrow microenvironment, researchers will be able to develop effective preventive strategies and implement early therapeutic interventions to hinder disease progression in individuals with severe VL.

## Author contributions

PSTV: Writing – review & editing. MdS: Writing – review & editing. CIB: Writing – review & editing. DBMF: Writing – review & editing. MSS: Writing – review & editing. JPBDM: Writing – review & editing. HT: Writing – review & editing. BML: Writing – review & editing.
